# An intravenous anesthetic drug-propofol, influences the biological characteristics of malignant tumors and reshapes the tumor microenvironment: A narrative literature review

**DOI:** 10.3389/fphar.2022.1057571

**Published:** 2022-11-25

**Authors:** Xueliang Zhou, Yanfei Shao, Shuchun Li, Sen Zhang, Chengsheng Ding, Lei Zhuang, Jing Sun

**Affiliations:** ^1^ Department of General Surgery, Ruijin Hospital, Shanghai Jiao Tong University School of Medicine, Shanghai, China; ^2^ Shanghai Minimally Invasive Surgery Center, Ruijin Hospital, Shanghai Jiao Tong University School of Medicine, Shanghai, China; ^3^ Shanghai Institute of Digestive Surgery, Ruijin Hospital, Shanghai Jiao Tong University School of Medicine, Shanghai, China/; ^4^ Department of Anesthesiology, Ruijin Hospital, Shanghai Jiaotong University School of Medicine, Shanghai, China

**Keywords:** malignant tumor, anesthetic drug, propofol, chemical properties, pharmacokinetic, biological characteristics, tumor microenvironment, postoperative prognosis

## Abstract

Malignant tumors are the second leading cause of death worldwide. This is a public health concern that negatively impacts human health and poses a threat to the safety of life. Although there are several treatment approaches for malignant tumors, surgical resection remains the primary and direct treatment for malignant solid tumors. Anesthesia is an integral part of the operation process. Different anesthesia techniques and drugs have different effects on the operation and the postoperative prognosis. Propofol is an intravenous anesthetic that is commonly used in surgery. A substantial number of studies have shown that propofol participates in the pathophysiological process related to malignant tumors and affects the occurrence and development of malignant tumors, including anti-tumor effect, pro-tumor effect, and regulation of drug resistance. Propofol can also reshape the tumor microenvironment, including anti-angiogenesis, regulation of immunity, reduction of inflammation and remodeling of the extracellular matrix. Furthermore, most clinical studies have also indicated that propofol may contribute to a better postoperative outcome in some malignant tumor surgeries. Therefore, the author reviewed the chemical properties, pharmacokinetics, clinical application and limitations, mechanism of influencing the biological characteristics of malignant tumors and reshaping the tumor microenvironment, studies of propofol in animal tumor models and its relationship with postoperative prognosis of propofol in combination with the relevant literature in recent years, to lay a foundation for further study on the correlation between propofol and malignant tumor and provide theoretical guidance for the selection of anesthetics in malignant tumor surgery.

## 1 Introduction

Malignant tumor is one of the diseases that seriously affect the human quality of life (Siegel et al., 2020). According to GLOBOCAN 2020 global cancer statistics, there were 9,958,133 deaths worldwide from malignant tumors in 2020 (Sung et al., 2021). Therefore, in-depth research into the pathogenesis of malignant tumors and the factors influencing their development has become a major scientific research direction for global health strategies. Currently, radiation therapy, chemotherapy, targeted therapy, and immunotherapy have improved the clinical outcome and extended the life of more patients with advanced malignant tumors, but surgical resection remains the most useful and effective treatment for solid malignant tumors. As a matter of fact, different surgical techniques and perioperative risk factors can influence the prognosis of tumor patients (Cuk et al., 2021).

The influence of perioperative anesthesia management on the postoperative prognosis of malignant tumors has increasingly come to light in the recent years. Studies have found that different anesthesia techniques and drugs can affect tumor recurrence and metastasis, resulting in different postoperative prognoses. Propofol exerts sedative-hypnotic effects by the means of chloride transport and γ-aminobutyric acid (GABA) receptors and is commonly used in terms of the induction and maintenance of general anesthesia, with the characteristics of rapid induction, rapid recovery, and few adverse effects (Feng et al., 2021). It is worth noting that many studies have demonstrated that propofol not only induces anesthesia and sedation but also alters the biological characteristics of the malignant tumors. It can suppress the malignant biological characteristics of tumor cells and promote apoptosis of tumor cells with anti-cancer activity (Eden et al., 2018). Interestingly, propofol has also been found to promote proliferation, invasion and metastasis of tumor cells in certain specific tumor types or conditions. In terms of therapeutic response, propofol modulates resistance to a number of chemotherapeutic agents and promotes drug sensitivity in tumor cells (Jiang et al., 2018). In addition, propofol can reshape the tumor microenvironment, reducing the degree of immunosuppression in the tumor microenvironment and inhibiting the signaling pathways that are involved in the development of inflammation and the production of inflammatory mediators, which has immune-activating and anti-inflammatory activity (Xu et al., 2020). Neoangiogenesis, hypoxia, and degradation of the extracellular matrix are important features of the tumor microenvironment that is also modulated by propofol. Clinical studies have shown that propofol is closely associated with postoperative prognosis for tumor patients, with the most extensive studies focusing on breast cancer (Wang et al., 2018). According to most studies, tumor patients receiving propofol-based anesthesia have a better prognosis than those receiving inhalation anesthesia. However, some retrospective studies have showed no significant difference between the prognosis of tumor patients undergoing propofol *versus* inhalation anesthesia, highlighting the need for large, multi-center, and prospective randomized controlled trials in the future.

**FIGURE 1:**
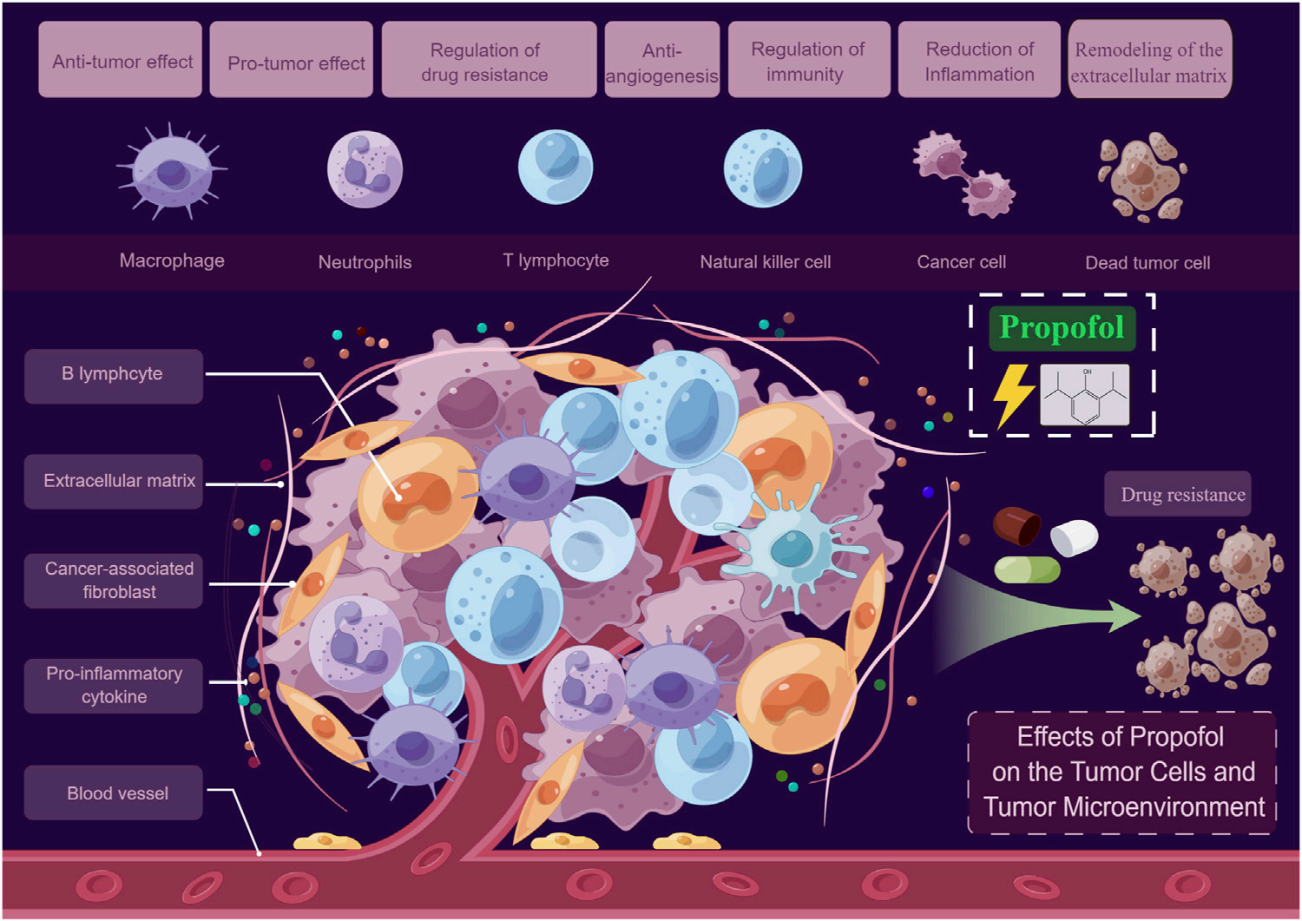
Effects of propofol on the tumor cells and tumor microenvironment. On one hand, propofol may directly act on tumor cells, exerting anti-tumor and pro-tumor effects, and regulating drug resistance. On the other hand, propofol may act indirectly on the tumor microenvironment by anti-angiogenesis, regulating immunity, reducing inflammation, and remodeling the extracellular matrix (By Figdraw).

Here we conducted a narrative literature review on propofol regarding its chemical properties, pharmacokinetics, clinical application and limitations, studies in animal tumor models as well as effects on the biological characteristics of malignant tumors, reshaping of the tumor microenvironment and postoperative prognosis, intending to lay a theoretical foundation for future large-scale prospective multi-center clinical trials and provide potential guidance for the precise selection of surgical anesthetics (Figure 1).

## 2 Chemical properties of propofol

**FIGURE 2:**
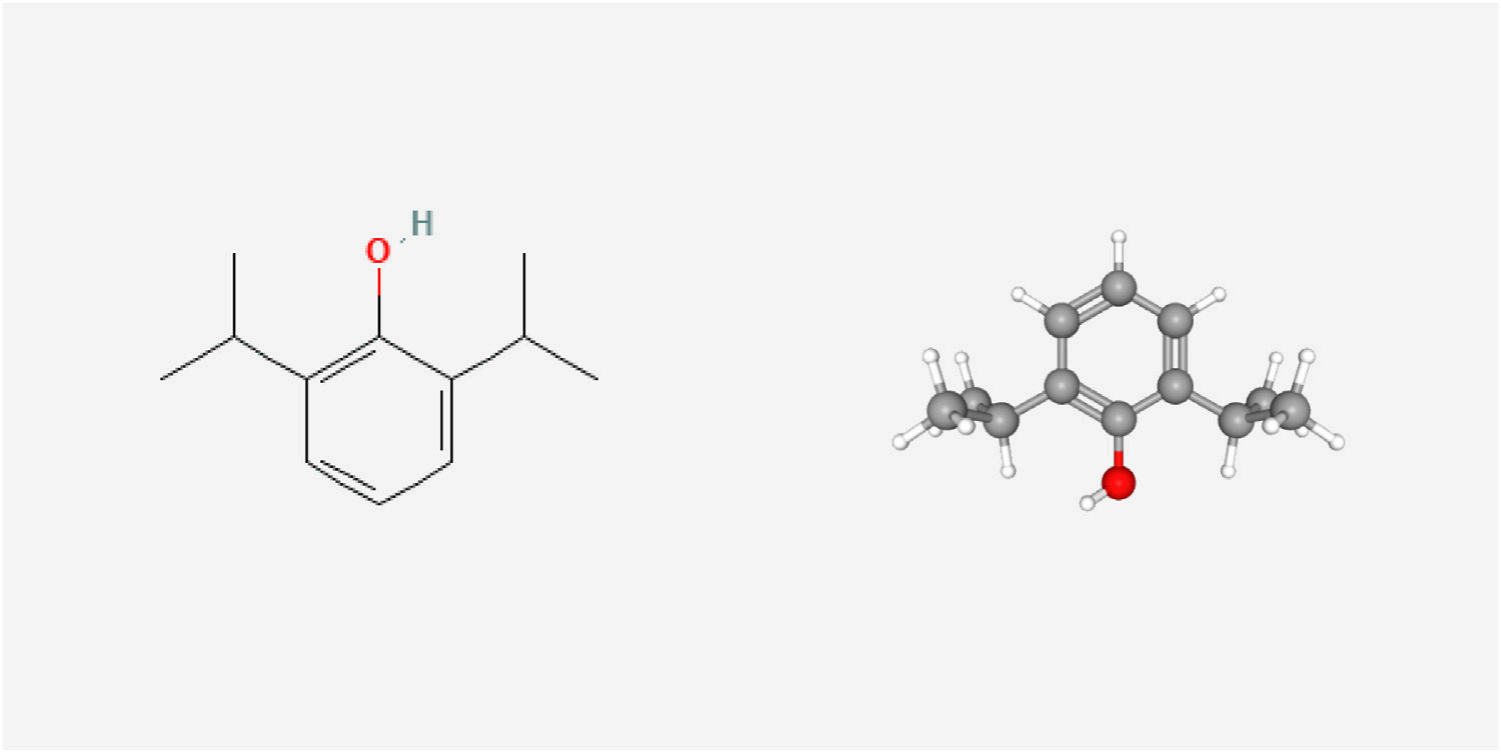
2D and 3D chemical structure of propofol.

Originally known as 2,6-diisopropyl phenol, propofol was discovered by Scottish chemist John B. Glen and used as an anesthetic. The 2D and 3D chemical structure of propofol is presented in Figure 2 (Cited from the PubChem database). Currently, propofol is currently the most widely used anesthetic drug in clinical practice, with the advantages of rapid onset of action, rapid recovery, rapid metabolic clearance and few adverse effects. In clinical practice, propofol fat emulsion is commonly used because it is a colorless or light-yellow liquid, almost insoluble in water but soluble in many organic solvents (Dinis-Oliveira, 2018). At present, it is widely believed that propofol has antioxidant effects. Studies have shown that the chemical structure of propofol contains phenolic hydrocarbon groups, which are similar to the known antioxidants 2, 6-di-tert-butylp-cresol and the endogenous antioxidant vitamin E. Propofol can directly react with oxygen free radicals to generate stable 2, 6-diisopropyl phenoxy group, which is to replace the highly active free radical with the low active free radical and reduce the lipid peroxidation cascade reaction triggered by the latter (Tsuchiya et al., 2010). Murphy et al. used electron rotational resonance spectroscopy to demonstrate that propofol acts as an antioxidant by reacting with free radicals to form phenoxy (Stratford and Murphy, 1997). Naohiro et al. (Kokita and Hara, 1996) found that small amounts of propofol in plasma could protect cell membranes by acting as an antioxidant, even when bound to plasma proteins. Several *in vivo* and *in vitro* studies have also shown that propofol can act as an antioxidant by rapidly and stably scavenging free radicals and inhibiting the process of lipid peroxidation (Li et al., 2012; Rosenfeldt et al., 2013).

It has been found that chemotherapeutic drugs such as formyl benzamide and chlorobenzene butyric acid have benzene ring or aromatic structures that contribute to their antitumor pharmacological effects (Wu et al., 2022). Propofol also exhibits antitumor effects and possesses a benzene ring structure, whereas other anesthetic drugs that do not exhibit antitumor effects, such as desflurane and sevoflurane, do not possess this ring structure. As a result, it has been speculated that the anti-tumor activities of propofol may be correlated with the benzene ring structure, but further research is needed to determine the exact relationship.

## 3 Pharmacokinetics of propofol

The pharmacokinetics of propofol is characterized by a three-compartment linear model. The three-compartment model refers to plasma, fast equilibrium tissue, and slow equilibrium tissue, characterized by rapid distribution and rapid elimination. Propofol is rapidly distributed in the whole body after intravenous injection. Within 40 s (single-arm cerebral circulation time), propofol can rapidly produce a slightly hypnotic effect. It is estimated that the half-life of blood-brain balance is about one to 3 min, which is probably the reason for the rapid induction of anesthesia (Wan Hassan et al., 2018). Propofol is highly lipophilic and is widely distributed in the brain and liver, followed by the heart, kidney, gastrointestinal tract, and fat tissue. The tissue concentration rapidly decreases 2 h after administration, indicating there is no apparent accumulation of propofol in tissue (Hüppe et al., 2020). However, a longer infusion of propofol resulted in a significant tissue accumulation, slowing down the decrease of circulating propofol, which subsequently increased the awakening time. Three phases are involved in the elimination of propofol. Phase I represents the rapid distribution of propofol with a half-life of 2–10 min; Phase II represents the elimination of propofol from the blood through metabolism with a half-life of 21–56 min; Phase III represents the return of propofol from poorly perfused tissue to the blood with the half-life of termination 200–300 min (Ji et al., 2020).

**FIGURE 3:**
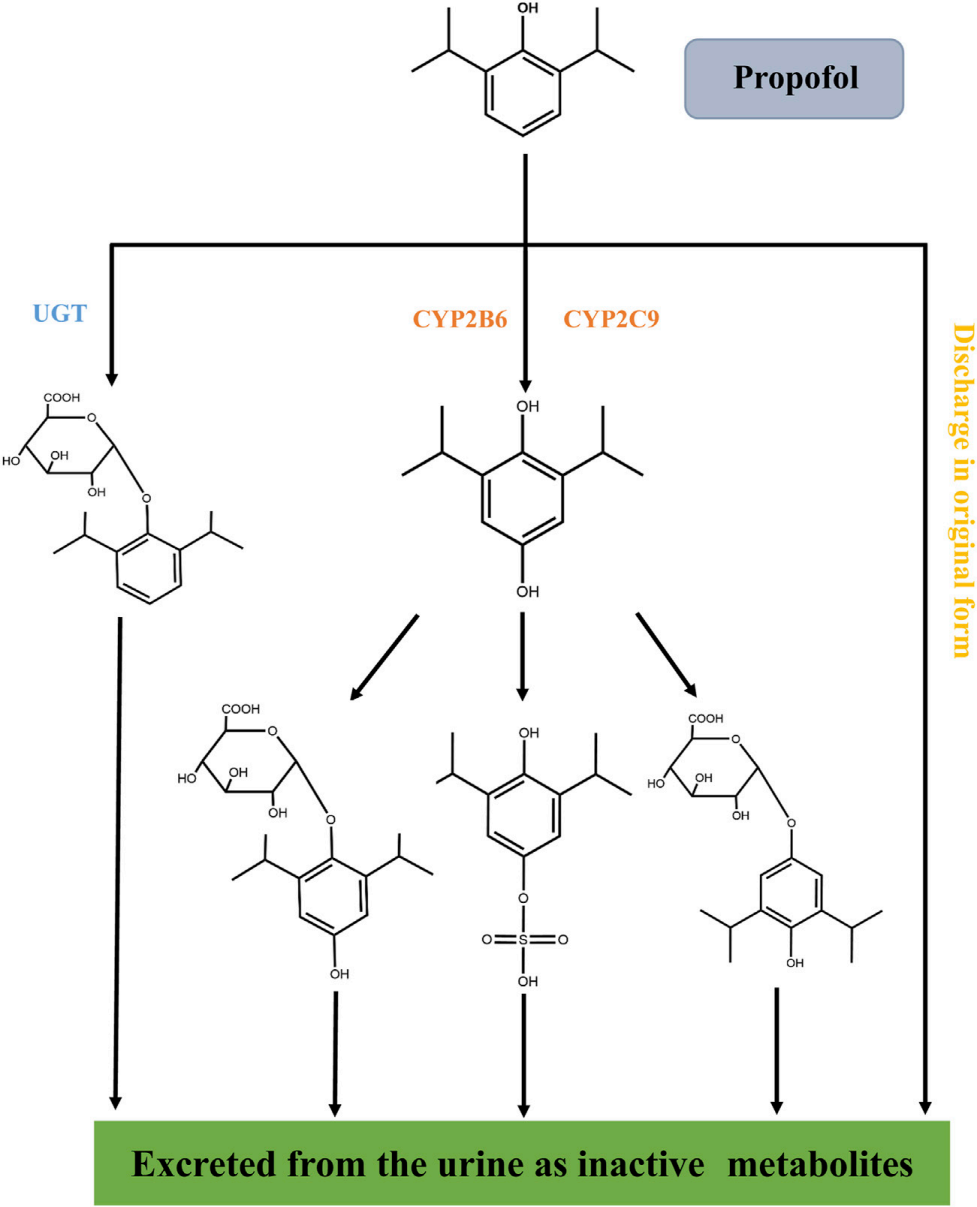
Metabolic process of propofol *in vivo*. Propofol is rapidly metabolized into inactive compounds in the liver through the hydroxylation of cytochrome P450 (CYP2B6 and CYP2C9) isomers and the UDP-glucuronosyhransferase (UGT) pathway and excreted by the kidney. Less than 1% of propofol is excreted in urine and 2% in feces.

Propofol is mainly metabolized in the liver (Figure 3). It is rapidly metabolized to inactive compounds through the hydroxylation of cytochrome P450 (CYP2B6 and CYP2C9) isomers and UDP-glucuronosyhransferase (UGT) pathway and eliminated by the kidney (Kodama et al., 2020). Less than 1% of propofol is excreted in urine and 2% in feces (Sandra et al., 2021).

Through the in-depth study of the pharmacokinetics of propofol, we can understand the absorption, distribution, and elimination process of propofol in the human body and the target organs where it exerts its pharmacological effects, which will further indicate the target organs and directions for the study of propofol. Propofol is highly lipophilic and protein-binding and is widely distributed in the liver, brain, gastrointestinal tract, and adipose tissue, so propofol tends to act in these organs. From a review of the literature, we found that propofol exerted a greater effect on liver cancer, gastric cancer, colorectal cancer and breast cancer, which is more consistent with the pharmacokinetic characteristics of propofol. Therefore, an in-depth understanding of the pharmacokinetics of propofol in the human body is of great significance for the rational use of drugs, the prediction of efficacy and toxicity, and the study of pharmacological effects.

## 4 Clinical application and limitations of propofol

### 4.1 Clinical application

Propofol is a short-acting intravenous anesthetic with the advantages of rapid onset, short duration of action, rapid elimination and low incidence of post-operative nausea and vomiting. It can depress the central nervous system and produce sedative and hypnotic effects, and is commonly used for induction and maintenance of anesthesia as well as sedation and analgesia. It is now commonly used for the induction and maintenance of general anesthesia (GA) and total intravenous anesthesia (TIVA), as well as for sedation during anesthesia, after surgery and in the intensive care unit (ICU).

#### 4.1.1 Total intravenous anesthesia

The most common types of general anesthesia are propofol-based total intravenous anesthesia (TIVA) and volatile drug-based inhalation anesthesia. Of course, in many cases a combination of intravenous and inhalation anaesthesia is also used. Total intravenous anesthesia is a method of administering anesthesia to patients using intravenous anesthetics and its auxiliary agents with the advantages of rapid induction, smooth anesthesia, no contamination and quicker awakening (Ramirez and Cata, 2021). Propofol has been the basis of TIVA, with the development of target-controlled infusion (TCI) system, TCI has the advantages of fast adjustment speed, strong controllability and stable anesthesia depth, which is conducive to the personalized and refined administration of propofol, and can effectively avoid the sedation or anesthesia of too deep or too shallow due to individual differences and changes in surgical stimulation intensity (Anderson and Bagshaw, 2019).

#### 4.1.2 Sedation in ICU

At present, sedation and analgesia has become one of the important treatment methods in ICU, which can reduce stress, reduce the body oxygen consumption, make the patient in a comfortable state, reduce the accident of extubation and is conducive to prevent accidents and the recovery of the patients. Propofol is a fast-acting, short-acting, fast-recovery intravenous anesthetic. It can achieve good sedative effect under the premise of analgesia and is suitable for short-term sedation in ICU (Garcia et al., 2021).

#### 4.1.3 Application in outpatient endoscopic surgery

In recent years, with the continuous development of endoscopic techniques and the increased demand for painless endoscopy, propofol is also being used for various outpatient endoscopic surgeries such as painless gastrointestinal endoscopy and tracheoscopy. Propofol is becoming increasingly popular in sedation for gastrointestinal endoscopy due to its unique pharmacokinetic properties, predictable recovery process and rapid recovery (Goudra et al., 2021).

### 4.2 Limitations in clinical practice

Currently, propofol is commonly used clinically as a fat emulsion formulation, and a number of problems remain in its clinical application. Apart from producing pharmacological adverse effects such as dose-related blood pressure drop, heart rate decreases and apnea, others are mainly associated with propofol in fat emulsion formulations, such as injection site pain, thrombophlebitis, hypertriglyceridemia, potentially fatal bacterial infections, rupture and blockage of the infusion line during prolonged infusion, propofol infusion syndrome (PRIS) and allergic reactions, *etc.*

#### 4.2.1 Decreased blood pressure and respiratory depression

Propofol has a cardiovascular depressant effect and causes hypotension associated with reduced peripheral vascular resistance, reduced cardiac preload, reduced sympathetic activity and myocardial contractility (Doğanay et al., 2018). Studies have confirmed that propofol-induced hypotension is related to the rate of injection, the dose injected and the effect on the central nervous system. The drop in blood pressure induced by propofol usually lasts for a short period of time and its cause of persistent hypotension is most often seen in elderly, female, poor general condition or in patients on concomitant morphine-like drugs.

In clinical use, propofol is highly likely to cause respiratory depression. Even the induced dose of propofol can cause slower respiration, reduced tidal volume and even greater degree and frequency of apnea than other intravenous anesthetics of the same type (Jiang et al., 2021).

#### 4.2.2 Injection site pain

Injection site pain can occur in both adults and children, this pain can be immediate or delayed. This pain is due to the activation of the plasma pancreatic Vaso peptide system by propofol and the subsequent production of bradykinin, which is not the only factor causing the pain (Miniksar, 2022). Pain caused by intravenous propofol is not a very serious complication, but pain can be an important source of excessive stress in patients during surgery and an important limiting factor for ideal anaesthesia.

#### 4.2.3 Hypertriglyceridemia

Propofol is mainly given as an emulsion, and prolonged infusion is accompanied by elevated lipid levels. The long-chain triglycerides in the emulsion are the main factor causing elevated lipid levels (Corrado et al., 2020). In addition. The ability to metabolize and remove fat is reduced by changes in the enzymatic systems involved in lipid clearance and metabolism, which may result in hypertriglyceridemia due to the increased fat load and metabolic disturbances associated with the administration of propofol in stressful situations.

#### 4.2.4 Propofol infusion syndrome

PRIS is a dangerous adverse reaction in the use of propofol and its treatment success rate is low. It was found that when propofol dose > 4 mg/(kg h) and infusion time >48 h may lead to PRIS. The main manifestations are unexplained cardiac arrhythmias, metabolic acidosis, hyperkalemia and cardiomyocyte lysis, which eventually develop into severe heart failure and even lead to the death (Hemphill et al., 2019). Triggers of PRIS include low age, severe central nervous system disease, excessive intake of glucocorticoids or exogenous catecholamines and inadequate carbohydrate intake.

## 5 Effect of propofol on biological characteristics of malignant tumor cells

Surgical resection is the primary treatment for solid malignancies. Numerous studies have demonstrated that the choice of anesthetic is closely associated with the postoperative prognosis of tumor patients. As a commonly used anesthetic, propofol not only has an anesthetic effect but also may act on tumor cells directly, which affects the biological characteristics of tumor cells, including anti-tumor effect, pro-tumor effect, and regulation of drug resistance.

### 5.1 Anti-tumor effect

**FIGURE 4:**
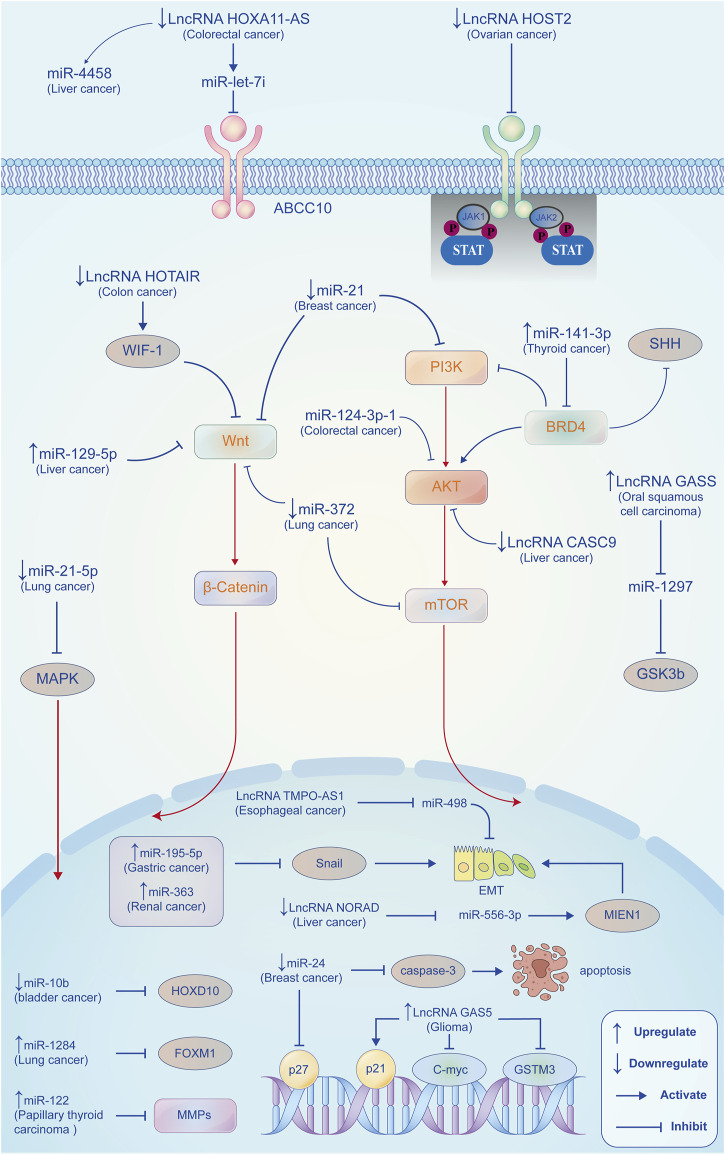
Propofol exerts anti-tumor effects by regulating miRNAs/lncRNAs. In different tumor types, propofol regulates the expression of miRNAs (proto and anti-oncogenes) and lncRNAs to regulate different tumorigenesis and development-related signaling pathways, genes, and proteins expressions, such as PI3K/AKT, Wnt/β-catenin, MMPs, P27, and P21, thus inhibiting the proliferation, invasion, and metastasis of tumor cells, reducing EMT process and promoting apoptosis.

The progression of malignant tumors is regulated by a variety of key factors both *in vitro* and *in vivo*. It has been shown that the anti-tumor effects of propofol are mainly through the regulation of microRNAs (miRNAs) and long non-coding RNAs (lncRNAs) expression to target multiple signaling pathways, oncogenes and functional proteins (Figure 4). Interestingly, in recent studies, propofol can also inhibit the progression of tumors by attenuating the function of tumor stem cells and regulating metabolic reprogramming.

#### 5.1.1 Propofol and miRNAs

MiRNAs are a large family with distinctive structural and functional characteristics and are small non-coding single-stranded RNAs. Mature miRNAs are about 20–24 bases in length and are formed from a segment of single-stranded RNA precursor of 70–90 bases in length with a hairpin loop structure processed by an RNA enzyme called Dicer (Budakoti et al., 2021). Mature miRNAs inhibit translation or induce degradation of target gene mRNA molecules mainly by forming RNA-induced silencing complexes (RISCs) through complementary matching with their 3′-UTRs (Slack and Chinnaiyan, 2019). Numerous studies have demonstrated that miRNAs are closely related to tumorigenesis and development, affecting tumor differentiation, proliferation, invasion, metastasis, and drug resistance through controlling the expression of target genes, as well as their cell cycle and apoptosis process, thus serving as anti-oncogenes. (He et al., 2020). Therefore, miRNAs can be used as biological markers for diagnosis and prognosis prediction, as well as valuable therapeutic targets for tumors.

Some miRNAs, as proto-oncogenes, can negatively regulate anti-oncogenes, promote malignant transformation of normal cells, enhance proliferation, and differentiation and inhibit apoptosis of tumor cells. Propofol can suppress tumor cell proliferation, migration and invasion by downregulating proto-oncogenic miRNAs (Table 1). In bladder cancer, Qi et al. (2019a) found that propofol downregulated the miR-10b expression and increased the expression of its target gene HOXD10, exerting tumor-suppressive effects. Propofol suppresses the malignant biology of lung cancer cells through down-regulation of miRNAs. Wu et al. (2020) have showed that propofol inhibited NSCLC cell viability by downregulating the miR-21-5p/MAPK10 axis. Sun and Gao (2018) demonstrated that propofol suppressed the proliferation and migration of lung cancer A549 cells, partly due to downregulation of miR-372. In breast cancer, propofol could promote apoptosis of tumor cells by upregulating P27 and cleaved caspase-3 expression *via* downregulating miR-24 (Yu et al., 2018). Du et al. (2019) also found that propofol inhibited the activation of PI3K/AKT and Wnt/β-catenin signaling pathways through down-regulation of miR-21 expression, thereby reducing cell proliferation of breast cancer.

**TABLE 1:** Propofol inhibits tumors by regulating miRNAs/lncRNAs.

MiRNA/LncRNA	Regulation	Cancer types	Targets	Mechanism	Ref
MiRNA	**Downregulation**	Bladder cancer	miR-10b	miR-10b/HOXD10	Qi et al. (2019a)
		Lung cancer	miR-21-5p	miR-21-5p/MAPK10	Wu et al. (2020)
		Lung cancer	miR-372	Suppressed Wnt/β-catenin and mTOR signaling pathways	Sun and Gao, (2018)
		Breast cancer	miR-24	Upregulating p27 and cleaved caspase-3 expression	Yu et al. (2018)
		Breast cancer	miR-21	Suppressed Wnt/β-catenin and PI3K/AKT signaling pathways	Du et al. (2019)
	**Upregulation**	Gastric cancer	miR-195-5p	miR-195-5p/snail	Liu et al. (2020)
		Renal cancer	miR-363	Reduced the expression of Snail1	Shi et al. (2021)
		Lung cancer	miR-1284	Reduced the expression of FOXM1	Liu and Liu, (2018)
		Papillary thyroid carcinoma	miR-122	Reduced the expression of MMP2 and MMP9	Li et al. (2020a)
		Colorectal cancer	miR-124-3p	Reduced the expression of AKT3	Li et al. (2020b)
		Liver cancer	miR-219-5p	Suppression of GPC3-mediated Wnt/β-catenin signaling activation	Gong et al. (2019)
		Thyroid cancer	miR-141-3p	Suppressing SHH and PI3K/AKT signaling pathways *via* the miR-141-3p/BRD4 axis	Zhang et al. (2021a)
LncRNA		Ovarian cancer	lncRNA HOST2	Suppressing JAK2/STAT3 signaling pathway	Shen et al. (2021)
		Liver cancer	lncRNA CASC9	Suppressing Akt/mTOR signaling pathway	Chang et al. (2022)
		Colon cancer	lncRNA HOTAIR	Activating WIF-1 and Suppressing Wnt pathway	Zhang et al. (2020a)
		Glioma	lncRNA GAS5	Increasing the expression of P21, decreasing the expression of c-myc and GSTM3	Cheng et al. (2022)
		Oral squamous cell carcinoma	lncRNA GAS5	FoxO1-GAS5-miR-1297-GSK3b axis	Gao et al. (2019)
	**Interactions between lncRNA and miRNA**	Colorectal cancer	lncRNA HOXA11-AS	lncRNA HOXA11-AS/miR-let-7i/ABCC10	Ren and Zhang, (2020)
		Liver cancer	lncRNA HOXA11-AS	lncRNA HOXA11-AS/miR-4458	Song et al. (2020)
		Liver cancer	LncRNA NORAD	lncRNA NORAD/miR-556-3p/MIEN1	Liu et al. (2021a)
		Esophageal cancer	lncRNA TMPO-AS1	lncRNA TMPO-AS1/miR-498	Gao et al. (2020)

Some other miRNAs can also act as anti-oncogenes, antagonizing proto-oncogenes, inhibiting proliferation, differentiation, migration, and invasion, as well as promoting apoptosis of tumor cells. Studies show that propofol can inhibit tumor progression and improve prognosis by up-regulating anti-oncogenic miRNAs (Table 1). The epithelial-mesenchymal transition (EMT) is the conversion of polar epithelial cells into mesenchymal cells with the capacity for invasive metastasis, which is closely related to tumor development. Propofol can inhibit the EMT process by regulating miRNA. Liu et al. (2020) reported that propofol could inhibit snail expression by promoting the activity of miR-195-5p, thereby reducing EMT, migration and invasion of gastric cancer cells. Shi et al. (2021) revealed that propofol could upregulate the expression of miR-363 and decrease the expression of Snail1 to inhibit the EMT process of kidney cancer cells. In lung cancer (Liu and Liu, 2018), propofol inhibits the proliferation and EMT process of lung cancer cells by upregulating the expression of miR-1284. In papillary thyroid cancer, Li et al. (2020a) reported that propofol upregulated miR-122 expression to suppress the invasion and the EMT process of tumor cells. Similarly, Propofol can target several important signaling pathways in tumors through upregulation of tumor suppression-related miRNAs. In colorectal cancer (Li et al., 2020b), propofol suppressed the malignant properties of colorectal cancer cells by upregulating miR-124-3p expression and downregulating AKT3 expression. Gong et al. (2019) found that miR-219-5p induced by propofol inhibited hepatocellular carcinoma proliferation and invasion by suppressing the Wnt/β-catenin signaling pathway mediated by GPC3. In thyroid cancer, propofol inhibited tumor cell proliferation, migration, and invasion by suppressing SHH and PI3K/AKT signaling pathways *via* the miR-141-3p/BRD4 axis (Zhang et al., 2021a). These studies indicate that miRNAs are pivotal therapeutic targets for tumors, and propofol can exert anti-tumor effects by regulating miRNAs expression, which further enriches the anti-tumor mechanism of propofol.

#### 5.1.2 Propofol and lncRNAs

Long non-coding RNA (lncRNA) is a kind of functional RNA molecule that cannot be translated into protein. They have mRNA-like structures and complex mechanisms of action. Numerous studies have demonstrated that lncRNAs play an important role in tumorigenesis and development by regulating epigenetic transmission, cell cycle and cell differentiation. Propofol can target various signaling pathways by regulating lncRNA expressions in tumor cells, such as the Wnt pathway, JAK2/STAT3 pathway, and Akt/mTOR pathway (Table 1). Shen et al. (2021) showed that propofol could inhibit the proliferation, invasion and apoptosis of ovarian cancer cells through the lncRNA HOST2/JAK2/STAT3 axis. Chang et al. (2022) reported that propofol exerted an anti-tumor effect by down-regulating lncRNA CAS9, thereby inhibiting the Akt/mTOR signaling pathway in hepatocellular carcinoma. In colon cancer, propofol promoted cell apoptosis and inhibited distant metastasis through the activation of WIF-1 and inhibition of Wnt signaling pathway by negatively regulating the expression of lncRNA HOTAIR (Zhang et al., 2020a). GAS5 is a FoxO1-activated long noncoding RNA that can be regulated by propofol. In glioma, Cheng et al. (2022) showed that propofol inhibited the growth and migration of glioma cells through upregulation of the lncRNA GAS5. Gao et al. (2019) found that lncRNA GAS5 promoted the apoptosis of propofol-induced oral squamous cell carcinoma by regulating the miR-1297-GSK3β axis.

It is worth noting that both lncRNAs and miRNAs are non-coding functional RNAs with epigenetic regulation, which are closely related to the malignant biological characteristics of tumor cells. LncRNAs can bind to miRNAs and affect their functions, while miRNAs can regulate the stability of LncRNAs. Numerous studies have indicated that propofol could exert its anti-tumor activity by modulating the interaction between lncRNAs and miRNAs. Among which, lncRNA HOXA11-AS is currently more widely reported. Ren and Zhang (2020) reported that propofol could promote apoptosis in colorectal cancer cells by reducing the inhibitory effect of HOXA11-AS on miR-let-7i. In hepatocellular carcinoma, Song et al. (2020) revealed that propofol downregulated the expression of lncRNA HOXA11-AS to enhance the expression of miR-4458 and inhibit the malignant biological behavior of tumor cells. In addition, propofol can also regulate the interaction between other lncRNAs and miRNAs, such as lncRNA NORAD/miR-556-3p/Migration and Invasion Enhancer 1 (MIEN1) axis (Liu et al., 2021a) and lncRNA TMPO-AS1/miR-498 axis (Gao et al., 2020). Therefore, an in-depth study of the interaction between lncRNAs and miRNAs is essential to understanding the mechanism of the anti-tumor effects of propofol.

#### 5.1.3 Attenuating the function of tumor stem cells

Tumor stem cells are cells in tumors that have the ability to self-renew and generate heterogeneous tumor cells, which play an important role in tumor survival, proliferation, metastasis and recurrence, and are also a cause of drug resistance (Richard et al., 2021). Recent studies have suggested that propofol could attenuate the function of tumor stem cells. Li et al. (2021) reported that propofol could inhibit the ability of bladder cancer stem cells to self-renew by targeting the hedgehog pathway, thereby inhibiting bladder tumor development and recurrence. In breast cancer, Zhang et al. (2019) found that propofol could reduce mammosphere formation in tumor stem cells *in vitro via* the PD-L1/Nanog pathway, thereby inhibiting cancer cell recurrence and metastasis. Another study showed that propofol inhibited the expression of circNOLC1 to attenuate tumor stem cell function *via* miR-365a-3p/STAT3 signaling pathway in breast cancer.

#### 5.1.4 Regulation of metabolic reprogramming

Metabolic reprogramming is an important feature of tumors. To adapt to the rapid and continuous proliferation of tumor cells, various metabolic pathways such as aerobic glycolysis, lipid biosynthesis, and glutamine metabolism will be reprogrammed in tumor cells, the most prominent of which is aerobic glycolysis, namely the Warburg effect (Gao et al., 2021a). The Warburg effect refers to the fact that under aerobic conditions, glucose in normal cells is oxidized in the mitochondria, whereas in cancer cells glucose does not enter the mitochondria but remains converted to lactate (Lebelo et al., 2019). Several studies have indicated that propofol can influence the metabolic reprogramming of tumor cells, with inhibition of glycolysis being the most commonly reported. Hu et al. (2019) reported that propofol could downregulated GLUT1 and MPC expression by downregulating HIF-1α and upregulating PEDF, thereby interfering with cancer cell glucose metabolism and inhibiting tumor progression. N-methyl-D-aspartate receptor (NMDAR), which controls Ca^2+^ flux, has recently been found to be associated with propofol-induced inhibition of glycolysis. Qi et al. found that (Qi et al., 2019b) propofol reduced intracellular Ca^2+^ concentration, CaMKII, AKT phosphorylation, and HIF-1α expression by inhibiting NMDA receptors, which in turn inhibited tumor and endothelial cell glycolysis levels and ultimately reduced tumor cell adhesion and metastasis. Chen et al. (2018) found that propofol inhibited aerobic glycolysis by inactivating the NMDAR-CAMKII-ERK signaling pathway in colorectal cancer. Circular RNAs (circRNAs) are a class of conserved non-coding RNAs with a closed-loop structure that is widely found in a variety of eukaryotes and are produced in higher eukaryotes by reverse splicing of multiple protein-coding genes through exons, which play an important modulatory role in tumorigenesis and progression. Propofol could inhibit glycolysis in tumor cells *via* the regulation of the circRNAs expression. Qu et al. (2022) found that propofol inhibited glycolysis in ovarian tumors by inhibiting the circular RNA-zinc finger RNA binding protein (circ-ZFR)/mir-212-5p/superoxide dismutase 2 (SOD2) axis. In lung cancer, propofol disrupted cell carcinogenesis and aerobic glycolysis by regulating circRNA transcriptional adaptor 2A (circTADA2A)/miR-455-3p/forkhead box M1 (FOXM1) axis (Zhao et al., 2020).

The presence of lipid-metabolism-related molecules and signaling cascades may contribute to tumor progression. However, no systematic studies have been published showing the specific mechanisms by which propofol regulates the reprogramming of lipid metabolism in tumor cells, but some basic and clinical researches reveal that propofol indeed affects lipid metabolic processes *in vivo*. Zhang et al. (2022a) found that propofol could promote glucagon-regulated gluconeogenesis and accelerate fatty acid beta-oxidation *via* the CREB/PGC-1α signaling pathway. A metabolomics study found that propofol significantly increased the ratio of saturated fatty acids to total fatty acids (SFA_FA), very large VLDL free cholesterol content (XL_VLDL_FC), and very large HDL triglyceride content, and slightly increased serum total triglyceride levels (Nummela et al., 2022). The above studies provide a new strategy for propofol to exert anti-tumor effects by regulating metabolic reprogramming.

### 5.2 Pro-tumor effect

Currently, numerous studies have found that propofol could inhibit the proliferation, differentiation, migration and invasion of tumor cells, mainly exerting anti-tumor effects. However, some studies have also confirmed that propofol can also promote tumor cell proliferation and migration in certain tumor types under certain conditions. In oral squamous carcinoma, propofol (Li et al., 2020c) promoted the migration of tumor cells *via* the upregulation of the SNAI1 expression. Another study in breast cancer also confirmed that propofol could induce the proliferation of breast cancer cells *via* downregulation of p53 protein and promote the invasion and migration of tumor cells *via* the activation of the Nrf2 signaling pathway (Meng et al., 2017). The adhesion of circulating tumor cells to vascular endothelial cells is a crucial factor in the development of solid tumor metastasis. Liu et al. (2021b) found that propofol could activate the GABA receptor in tumor cells and reduce TRIM21, thereby increasing the expression of the cell adhesion-related protein Src and enhancing the adhesion and extension of tumor cells to vascular endothelial cells, thus promoting tumor metastasis in the lung of mouse models. Therefore, an in-depth investigation into the mechanism of propofol-promoting tumor cell proliferation and migration will further enrich the pharmacological effects and the scope of application of propofol, and also raises more attention to the selection of propofol in clinical practice.

### 5.3 Regulation of drug resistance

Due to the loss of the best surgical opportunity, chemotherapy often becomes the main treatment method to prolong the survival and improve the life quality of patients with unresectable distant metastases. In recent years, the diversity of chemotherapeutic drugs and treatment options has improved the survival rate of patients with advanced-stage cancer. However, the emergence of chemo-resistance is still a great challenge in the process of chemotherapy (Sadri Nahand et al., 2021). Studies have found that the use of propofol can regulate the chemo-resistance of tumors and improve the therapeutic effect of a variety of chemotherapy drugs. Several platinum-based chemotherapeutic agents, such as cisplatin (DDP) and oxaliplatin, have become the first-line chemotherapeutic agents for many malignant tumors based on the abilities to block DNA replication and inhibit mitosis. Huang et al. (2020a) found that propofol could enhance the DDP sensitivity of lung cancer cells *via* inhibition of the Wnt/β-catenin signaling pathway. According to Zhang et al. (2020b), propofol inhibited autophagy, promoted cisplatin sensitivity, and suppressed tumor progression *via* modulating the lncRNA MALAT1/miR-30e/ATG5 axis in gastric cancer. Sun et al. (2020) indicated that propofol restrained DDP resistance by downregulating miR-374a and upregulating FOXO1. In liver cancer, it is shown that propofol enhanced the lethality of cisplatin on tumor cells by up-regulating miR-195-5p (Gao and Zhang, 2022). Paclitaxel (PTX) and docetaxel, which are taxane-based chemotherapy drugs, boost the polymerization of microtubule proteins and prevent their depolymerization in order to block the mitotic process and induce apoptosis, which are used together with other anti-cancer drugs in the treatment of a variety of tumors. Yang et al. (2021) showed that propofol enhanced the sensitivity of prostate cancer cells to PTX by reducing the expression of HOTAIR, which promoted apoptosis of cancer cells. In prostate cancer, propofol also reversed hypoxia-induced docetaxel resistance by inhibiting HIF-1α to prevent epithelial-mesenchymal transition (Qian et al., 2018). 5-Fluorouracil (5-FU) is an anti-metabolic chemotherapeutic agent that inhibits the synthesis of DNA and RNA. Yang et al. (2022) found that propofol-induced apoptosis ameliorated 5-FU resistance in oral squamous cell carcinoma cells by the reduction of amphiregulin expression and secretion. Considering that propofol can modulate glucose and lipid metabolism in tumor cells, we speculate that it may modulate resistance to anti-metabolic chemotherapeutic drugs *via* metabolic pathways.

In recent years, the emergence of new therapeutic agents such as targeted therapy and immunotherapy has provided new strategies for the treatment of malignant tumors. Trastuzumab, a monoclonal antibody against Her-2, is a valuable targeted therapeutic agent. Tian et al. (2020) found that propofol could epigenetically regulate trastuzumab resistance *via* the IL-6/miR-149-5p axis in breast cancer. However, no studies have been reported on whether propofol modulates the sensitivity of immunotherapy drugs, and this deserves further investigation. The above studies suggest that propofol plays an important role in modulating systemic treatment resistance, which will provide new therapeutic strategies for the clinical reversal of drug resistance in cancer treatment.

## 6 Effect of propofol on tumor microenvironment

**FIGURE 5:**
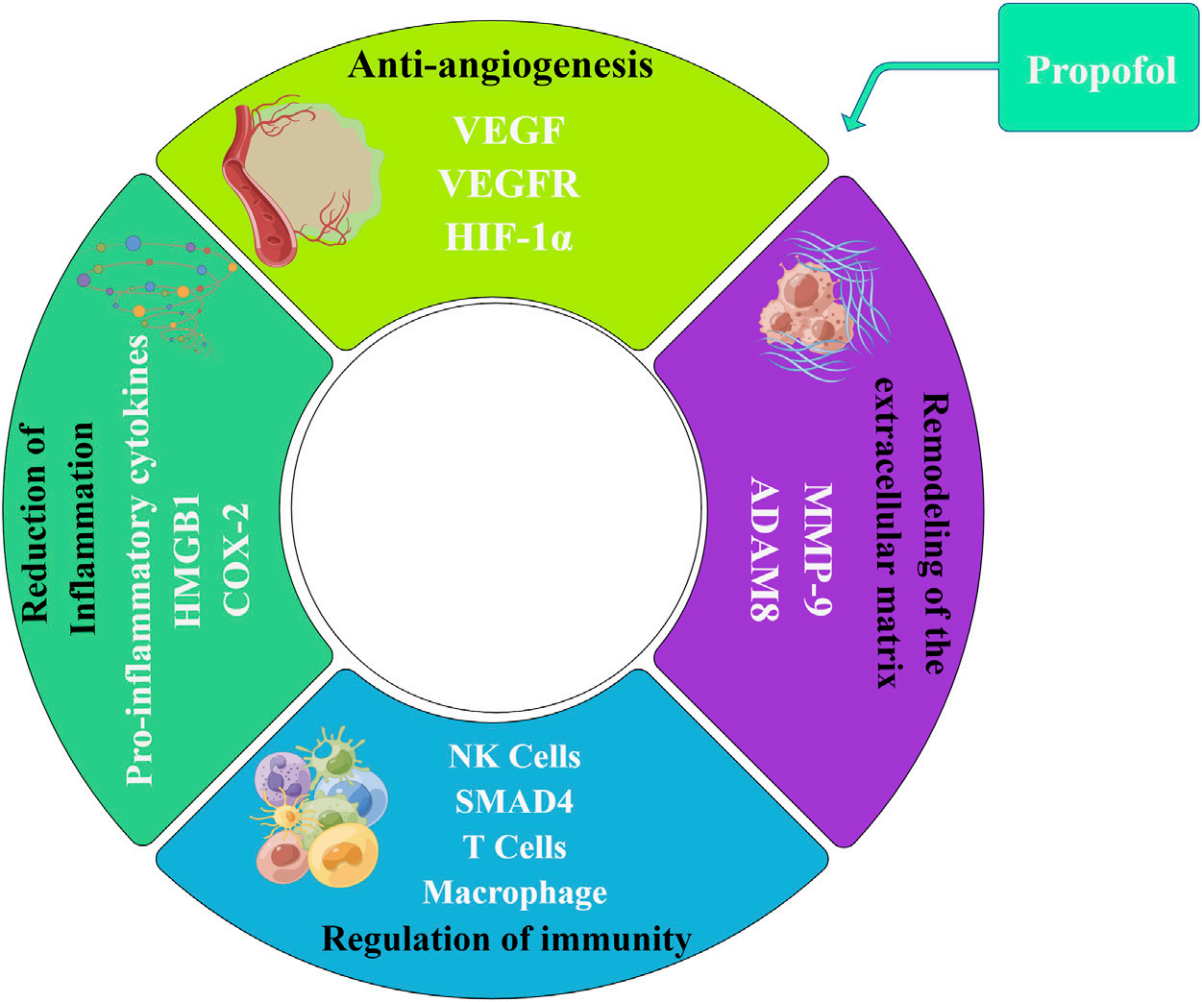
Effect of propofol on the tumor microenvironment. Propofol can play a role in reshaping the tumor microenvironment, including anti-angiogenesis, regulation of immunity, reduction of inflammation, and remodeling of the extracellular matrix, indirectly affecting the biological characteristics of tumor cells. Propofol can inhibit the expression of VEGF/VEGFR and play an anti-angiogenesis role. Propofol regulates immunity by affecting the infiltration and activity of a variety of immune cells, such as T cells, NK cells, and macrophages. Propofol can reduce inflammation via inhibiting the release of pro-inflammatory cytokines and targeting the expression of HMGB1 and COX-2 inflammatory proteins. In addition, propofol can target the expression of MMP-9 and ADAM8 to remodel the tumor extracellular matrix.

The tumor microenvironment (TME) can be described as a highly complex system, which is composed of tumor cells, interstitial cells around tumor cells (such as immune cells, fibroblasts, adipocytes, and endothelial cells), extracellular matrix (ECM), and signal molecules (such as cytokines and chemokines). The two-way interaction between tumor cells and TME affects the tumorigenesis and progression at multiple levels. Tumor cells can change TME to produce a suitable living environment, and TME can in turn affect the behavior of tumor cells (Deepak et al., 2020). With the increasing understanding of the interaction between tumor cells and TME, TME may become the target of novel drugs (Xiao and Yu, 2021). Studies have shown that propofol can play a role in reshaping the tumor microenvironment, including anti-angiogenesis, regulation of immunity, reduction of inflammation and remodeling of the ECM, indirectly affecting the biological characteristics of tumor cells (Figure 5).

### 6.1 Anti-angiogenesis

Tumor growth and metastasis are inseparable from the formation of neovascularization. When the tumor volume continues to increase and the center of tumor necrosis occurs as a result of hypoxia, tumor cells will release pro-angiogenic factors to stimulate peripheral neovascularization (Dzhalilova and Makarova, 2021). Blood circulation not only provides the necessary oxygen and nutrients for tumor growth but also acts as an important way for tumor cells to metastasize (Wang et al., 2021a). Currently, anti-angiogenesis has become an important targeted therapy strategy for maximum disease control of unresectable metastatic solid tumors. More and more studies have indicated that propofol can suppress angiogenesis and play an anti-tumor role. A clinical trial found that total intravenous anesthesia with propofol during radical lung cancer surgery significantly reduced serum concentrations of VEGF and other angiogenesis-related factors in patients, favoring the anti-angiogenesis effect (Sen et al., 2019). Wang et al. (2021b) found that propofol could inhibit tumor angiogenesis by targeting VEGF/VEGFR and mTOR/eIF4E signaling, thus exerting anti-cancer activity. In human esophageal cancer EC-1 cells. Guo et al. (2015) demonstrated that propofol downregulated S100A4 expression levels to inhibit proliferation, invasion, and angiogenesis, as well as promote apoptosis. Chen et al. (2017) found that propofol could inhibit the expression of VEGF in pancreatic cancer cells *in vitro* and *in vivo*, possibly by inhibiting the NMDA receptor. Hypoxia is an important feature of the tumor microenvironment and is an important cause of tumor neovascularization. When the oxygen partial pressure of the tumor microenvironment is reduced, hypoxia-inducible factors (HIF) are activated to regulate primary transcriptional adaptation to the hypoxic microenvironment (Li et al., 2020d). Yang et al. (2017) found that HIF-1α was upregulated at both the gene and protein levels in LPS-treated NSCLC tumor cells compared to normal tissue. Propofol could inhibit the upregulation of HIF-1α expression and reactive oxygen species (ROS) production in NSCLC tumor cells induced by LPS, suppressing the expression of VEGF, promoting tumor cell apoptosis as well as inhibiting invasion and metastasis. The above studies give strong support for the anti-angiogenic effect of propofol and its anti-cancer activity, providing new ideas on the effects and mechanisms of propofol on tumor neovascularization, as well as providing more options for the use of propofol and targeted tumor angiogenesis therapy.

### 6.2 Regulation of immunity

The development of the tumor is closely related to the immune microenvironment, which is often in an immunosuppressive status providing a favorable environmental basis for tumor growth and proliferation, immune escape, and acquired drug resistance (Pansy et al., 2021). Some studies have shown that propofol increases immune cell infiltration in the immune microenvironment and is closely associated with NK cell activity. Zhou et al. (2018) found that the use of propofol in esophageal cancer surgery can significantly enhance the toxicity of NK cells to cancer cells and enhance the killing activity of NK cells. Liu et al. (2018) collected 20 colorectal patients and isolated NK cells by screening and found that the expression of NK cell killing effector molecules was significantly increased in colorectal patients after treatment with propofol, indicating that propofol has the effect of enhancing NK cell killing activity. Another study compared propofol with sevoflurane anesthesia and showed that propofol could enhance the cytotoxicity of NK cells by up-regulating SMAD4 in gastric cancer surgery (Ai and Wang, 2020). However, there is no consensus on whether propofol can improve or reverse microenvironmental immunosuppression. By performing an immunoassay on 201 patients who randomly received propofol or sevoflurane anesthesia, Oh et al. (2018) found no significant differences in the infiltration levels of natural killer cells, cytotoxic T cells, cytokines, neutrophils, and lymphocytes between the two groups, indicating that propofol has few effects on the tumor immune microenvironment. Subsequently, the team conducted a prospective randomized trial of propofol on immune cell expression profiles in colorectal cancer patients. The results showed that propofol was not superior to sevoflurane in the alleviation of the suppression of immune cells in colorectal cancer surgery (Oh et al., 2022). Another randomized controlled trial on the effect of volatile anesthesia (sevoflurane) *versus* intravenous anesthesia (propofol) on immunosuppression in renal cancer also showed that propofol was not effective in improving the immunosuppressive state of the tumor microenvironment (Efremov et al., 2020). Therefore, the regulatory effects of propofol on the immune system, including immune cell infiltration and expression of immune checkpoints, are still unclear and need to be further investigated, which will enrich the immunomodulatory role of propofol and provide a new direction for tumor immunotherapy.

### 6.3 Reduction of inflammation

Inflammatory infiltrating cells interact with tumor cells through the release of mediators such as pro-inflammatory cytokines, constituting a complex tumor inflammatory microenvironment (McLaughlin et al., 2020). A variety of signaling pathways are activated by inflammatory stimulation, leading to an increase in oxidative enzyme activity, resulting in DNA and mitochondrial damage, and, ultimately, causing tumorigenesis (Atretkhany et al., 2016).

Studies have confirmed that propofol inhibits the release of pro-inflammatory cytokines and reduces inflammation in microenvironment. In the non-tumor tissue microenvironment, Liu et al. (2017a) showed that propofol attenuates the upregulation of pro-inflammatory cytokines in microglia by suppressing the NF-κB/p38 MAPK pathway activation. Ma et al. (2018) found that propofol increased ABCA1 expression and inhibited the production of pro-inflammatory cytokines in a LncRNA LOC286367-dependent manner. In the colorectal cancer microenvironment, Gao et al. (2021b) found that propofol might reduce the secretion of inflammatory cytokines by suppressing the activation of the NF-κB pathway *via* downregulating miR-155. The above study suggests that propofol might be used as a novel therapeutic strategy to alleviate the chronic inflammatory stimulation state of the tumor microenvironment.

The high mobility group protein B1 (HMGB1) is a well-conserved, highly adhesive nuclear protein involved in maintaining the nucleosome integrity and facilitating gene transcription, which is now considered to be an important late-stage inflammatory factor and is of greater clinical importance than early-onset rapid inflammatory factors such as TNF and IL-1 (Kim and Lee, 2020). Targeting HMGB1 is an effective strategy to improve the tumor inflammatory microenvironment. Li et al. (2020e) found that in papillary thyroid cancer, propofol decreased HMGB1 expression and inhibited tumor progression through downregulation of ANRIL. Jia et al. (2017) found that propofol could down-regulate LPS-stimulated HMGB1 expression in RAW 264.7 cell supernatants and reduce the releasing of LPS-stimulated IL-6, IL-8, and TNF-α.

Cyclooxygenase (COX), also known as prostaglandin endooxygenase reductase, is the key enzyme that catalyzes the conversion of arachidonic acid to prostaglandins (Timur et al., 2020). It consists of two isozymes, COX-1 and COX-2. Among them, COX-2 is inducible, which is the key to triggering the inflammatory response. Li et al. (2018) reported that propofol downregulates COX-2 expression to suppress proliferation and invasion of MCF-7 cells.

In conclusion, propofol can reduce inflammation in the tissue microenvironment by inhibiting the production and release of pro-inflammatory cytokines and targeting multiple inflammation-related proteins, which will lay a theoretical foundation for propofol to inhibit tumorigenesis by improving the tumor inflammatory microenvironment.

### 6.4 Remodeling of the extracellular matrix

The extracellular matrix (ECM) is a macromolecular substance that is synthesized and secreted by cells and distributed on the surface of cells or between cells, consisting of the basement membrane (BM) and the intercellular matrix. The ECM is linked to the inside and outside of the cell by membrane integrins, which involve cell survival, cell shape determination, cell differentiation modulation and cell migration control (Winkler et al., 2020).

Metalloproteinases (MMPs) are zinc-dependent endopeptidases that degrade almost all protein components of the extracellular matrix and disrupt the histological barrier. They are closely associated with tumor invasion and metastasis, and are an important biological marker for tumor invasion and metastasis (Dofara et al., 2020). Xu et al. (2013) found that propofol significantly downregulated the expression level of MMP-9 in esophageal cancer Eca-109 cells and inhibited invasion and metastasis.

The disintegrin and metalloprotease (ADAM) family are multifunctional proteins, consisting of eight structural domains including the metalloproteinase domain and deintegrin domain, which can be divided into membrane-anchored and secreted types (Camodeca et al., 2019). ADAM is closely related to tumor progression and is engaged in critical pathophysiological processes such as extracellular matrix degradation, cell signaling, and regulation of cell adhesion (Jones et al., 2016). In pancreatic cancer (Yu et al., 2020), propofol was found to downregulate the expression of ADAM8 and suppress tumor cells proliferation, invasion and migration. Yu et al. (2019) found that propofol could restrain the invasive and metastasis of tumor cells by upregulating miR-328 expression and suppressing the expression level of its target ADAM8 in pancreatic cancer.

## 7 Studies of propofol in animal tumor models

Advances in biomedical research often rely on the use of animal models as the basis for both experimental and clinical hypotheses. Animal studies can fill the gap between *in vivo* and clinical research. Propofol can not only affect the biological characteristics of tumor cells *in vitro*, but also play a role in animal tumor models. In hepatocellular carcinoma, Liu et al. (2017b) found that propofol could inhibit tumor growth and protein expression of MMP-2 and VEGF in xenograft model in a dose-dependent manner. Propofol can also activate AMPK to induce autophagy and thereby inhibit hepatocarcinogenesis and tumor volume in a xenograft mouse tumor model (Wang et al., 2020). Li et al. (2020f) conducted a study about the effect of propofol and sevoflurane on lung metastases in both syngeneic murine 4T1 and xenograft human MDA-MB-231 breast cancer models. For a long time, there is a lack of validated preclinical models for anesthesia studies, most of which do not use anesthesia itself as the primary endpoint to explore the effects of anesthesia on the tumor itself, the microenvironment and the postoperative prognosis. It is worth mentioning that Dubowitz et al. (2021) have successfully replicated key steps in clinical drug administration using propofol-TIVA anesthesia in a mouse model of mastectomy for breast cancer. The successful construction of this model will be of great help in studying the prognosis and mechanisms of action of anesthesia on patients with different cancer types.

## 8 Effect of propofol on postoperative prognosis

Perioperative factors such as inflammatory stimulation and metabolic changes can affect the prognosis of a tumor patient. In recent years, more and more attention has been paid to intraoperative anesthesia management, indicating that the different methods of anesthesia and drugs used have a significant impact on the prognosis of the tumor after surgery. Studies have shown that the use of propofol during operation is closely related to the disease-free survival rate and overall survival rate of various types of tumor patients. Therefore, the author combs and summarizes the clinical studies in the recent 5 years, hoping to lay a foundation for further study on the effect of propofol on postoperative prognosis (Table 2).

**TABLE 2:** The effects of propofol on postoperative prognosis of tumors.

Study type	Cancer type	Research design	Outcomes	Ref
Retrospective	Gastric cancer	Propofol vs*.* Sevoflurane	Improved survival	Zheng et al. (2018)
Retrospective	Gastric cancer	Propofol vs*.* Desflurane	Improved survival and reduced the risk of recurrence	Huang et al. (2020b)
Retrospective	Hepatocellular carcinoma	Propofol vs*.* Inhalation anesthetics	Decreased 2-year recurrence	Koo et al. (2020)
Retrospective	Hepatocellular carcinoma	Propofol vs*.* Desflurane	Better survival	Lai et al. (2019a)
Retrospective	Hepatocellular carcinoma	Propofol vs*.* Sevoflurane	Reduced mortality and recurrence	Meng et al. (2020)
Retrospective	Intrahepatic cholangiocarcinoma	Propofol vs*.* Desflurane	Improved survival and reduced the recurrence	Lai et al. (2019b)
Retrospective	Colon cancer	Propofol vs*.* Desflurane	Better survival	Wu et al. (2018)
Retrospective	Esophageal cancer	Propofol vs*.* Inhalation anesthetics	Better overall and recurrence-free survival	Jun et al. (2017)
Retrospective	Pancreatic cancer	Propofol vs*.* Desflurane	Improved survival	Lai et al. (2020a)
Prospective	Bladder cancer	Propofol vs*.* Sevoflurane	Improved disease-free Survival	Guerrero Orriach et al. (2020)
Retrospective	Prostate cancer	Propofol vs*.* Desflurane	Improved overall survival	Lai et al. (2020b)
Retrospective	Lung cancer	Propofol vs*.* Inhalation anesthetics	Better prognosis	Hayasaka et al. (2021)
Retrospective	Glioblastoma	Propofol vs*.* Desflurane	Better survival	Huang et al. (2021)
Retrospective	Breast cancer	Propofol vs*.* Sevoflurane	Better survival	Enlund et al. (2020)
Retrospective	Breast cancer	Propofol vs*.* Sevoflurane	Reduced local regional recurrence	(Zhang et al., 2021b), (Zhang et al., 2022b)
Retrospective	Breast cancer	Propofol vs*.* Desflurane	No difference	Huang et al. (2019)
Retrospective	Breast cancer	Propofol vs*.* Inhalation anesthetics	No difference	Yoo et al. (2019)
Retrospective	Breast cancer	Propofol vs*.* Sevoflurane	No difference	Shiono et al. (2020)
Prospective	Breast cancer	Propofol vs*.* Sevoflurane	No difference	Yan et al. (2018)
Retrospective	Gastric cancer	Propofol vs*.* Inhalation anesthetics	No difference	Oh et al. (2019)
Retrospective	Digestive tract tumor	Propofol vs*.* Inhalation anesthetics	No difference	Makito et al. (2020)
Retrospective	Glioblastoma	Propofol vs*.* Sevoflurane	No difference	Schmoch et al. (2021)
Retrospective	Glioma	Propofol vs*.* Sevoflurane	No difference	Dong et al. (2020)

Several retrospective clinical researches have shown that the use of propofol in anesthesia in tumor surgery can significantly improve the prognosis of patients. Propofol may improve the survival rate and reduce the recurrence and metastasis of patients with digestive tumors. In gastric cancer, Zheng et al. (2018) found that propofol-based anesthesia was correlated with better prognosis of patients undergoing gastrectomy. Huang et al. (2020b) found that propofol significantly improved the survival rate and reduced the risk of recurrence and metastasis in gastric cancer patients after a 5-year follow-up. In liver cancer, Koo et al. (2020) found that propofol can reduce the 2-year recurrence rate of early hepatocellular carcinoma, and significantly improve the prognosis of patients. Through a retrospective study on propofol and desflurane anesthesia in hepatectomy, Lai et al. (2019a) proved that propofol anesthesia is associated with a better prognosis. Meng et al. (2020) proved that propofol could significantly reduce the postoperative mortality and recurrence rate of hepatocellular carcinoma. Another study found that propofol could improve the postoperative survival rate and reduce the recurrence of intrahepatic cholangiocarcinoma (Lai et al., 2019b). Consistent with the above conclusion, the survival rate of propofol total intravenous anesthesia is higher than that of desflurane anesthesia in colon cancer surgery (Wu et al., 2018). Similarly, Jun et al. (2017) found that a better postoperative prognosis of esophageal cancer is closely related to propofol-based intravenous anesthesia. In pancreatic cancer, a retrospective analysis shows that propofol anesthesia can improve the postoperative survival rate of pancreatic cancer compared with desflurane anesthesia (Lai et al., 2020a). Propofol can also improve the prognosis of urinary tumors. Guerrero Orriach et al. (2020) found that compared with inhaled anesthetics and opioid analgesia, propofol intravenous anesthesia can improve the disease-free survival rate of patients with bladder cancer undergoing radical cystectomy. In radical prostatectomy for prostate cancer, propofol-based anesthesia also had a better survival rate than desflurane anesthesia (Lai et al., 2020b). Recently, Hayasaka et al. (2021) found that the use of propofol in early lung cancer surgery is correlated with a improved survival rate. Huang et al. (2021) found that propofol anesthesia was associated with better survival than desflurane anesthesia in glioblastoma surgery. In breast cancer, a multicenter retrospective analysis of 6,305 Swedish patients performed by Enlund et al. (2020) demonstrated that propofol improved the postoperative prognosis of patients better compared to sevoflurane. Zhang et al. (2021b); Zhang et al. (2022b) compared local-regional recurrence (LRR) in patients with invasive ductal carcinoma (IDC) under propofol-based paravertebral block-regional anesthesia (PB-RA) with LRR in patients undergoing inhalational general anesthesia (INHA-GA) with sevoflurane. The results suggested that propofol may be beneficial in reducing LRR in breast IDC patients compared to sevoflurane. The above studies suggest that propofol-based total intravenous anesthesia may contribute to better postoperative prognosis in many types of tumors.

However, some studies have found that intravenous anesthesia with propofol does not improve the postoperative prognosis of tumor patients compared with other volatile anesthesia. In breast cancer, Huang et al. (2019), Yoo et al. (2019), and Shiono et al. (2020) found that propofol anesthesia did not affect postoperative prognosis and survival of patients by retrospective analysis. A prospective, randomized and controlled study found that propofol/remifentanil total intravenous anesthesia was effective in inhibiting surgically induced VEGF-C release from breast cancer but did not appear to have a beneficial effect on short-term recurrence rates compared to sevoflurane inhalation anesthesia (Yan et al., 2018). In gastric cancer, Oh et al. (2019) found that propofol-based total intravenous anesthesia did not reduce overall or cancer-related mortality 1 year after surgery compared with inhalation anesthesia. In addition, Makito et al. (2020) found that propofol could not significantly improve the overall survival rate and recurrence-free survival rate of patients undergoing digestive tract tumor surgery (selective esophagectomy, gastrectomy, hepatectomy, cholecystectomy, pancreatectomy, colectomy, and rectal cancer surgery). Similarly, propofol-based intravenous anesthesia has no significant effect on the postoperative prognosis of glioblastoma (Schmoch et al., 2021) and glioma [139] in patients with nervous system tumors.

At present, the clinical research on the effect of propofol on the postoperative prognosis of tumors is mainly focused on retrospective analysis, which covers a wide range of tumor types, especially breast cancer. However, due to the limited sample size, the defects of retrospective study analysis, and the heterogeneity of clinical samples, the view that propofol can improve the postoperative prognosis and reduce recurrence and metastasis is worth further exploration and research. The conclusion is not yet consistent. We hope that in the future there will be more multi-center, large-scale prospective clinical trials with large samples to provide more theoretical evidence on whether propofol affects the postoperative prognosis of tumor patients and more precise guidance on the choice of anesthetic drugs in clinical practice.

## 9 Conclusion and prospect

Propofol is a commonly used clinical agent for total intravenous anesthesia. In this review, the chemical properties, pharmacokinetics, clinical application and limitations and studies in animal tumor models of propofol were summarized, as well as the effects on the biological characteristics of tumors and the reshaping of the tumor microenvironment were discussed in detail. *In vitro* and *in vivo* studies have shown that propofol exerts anti-tumor effects through mechanisms of action such as regulation of microRNA, lncRNA, stem cell function, and metabolic reprogramming. For some specific types of tumors or conditions, propofol can promote malignant biological behavior of tumors through a variety of mechanisms. Chemotherapy is an important treatment for malignant tumors, and chemo-resistance is one of the difficulties in oncology research and poor prognosis. Propofol regulates resistance to many common chemotherapeutic agents such as cisplatin, paclitaxel, and 5-fluorouracil, which provides a new strategy for reversing drug resistance. For the reshaping of the tumor microenvironment, propofol can act through anti-angiogenesis, regulation of immunity, reduction of inflammation, and remodeling of the ECM.

However, at present, the interaction between propofol and tumors is contradictory, and the conclusion is not yet consistent. Most studies found that propofol could suppress tumor proliferation, differentiation, metastases and promote apoptosis, and it is related to good postoperative prognosis. Nevertheless, some studies have demonstrated that propofol could promote the proliferation and migration of certain types of tumor cells, and it does not significantly improve the postoperative prognosis. Therefore, it is very important to clarify the mechanism of propofol on the occurrence and development of a malignant tumor and the relationship between propofol and postoperative prognosis. Meanwhile, we hope that there will be more large-scale prospective clinical trials with the multi-center, multi-sample, and multi-level to further explore the internal relationship between propofol and malignant tumors and guide more accurate intraoperative anesthesia management. Finally, it is worth emphasizing that this is a narrative review and the studies listed are not exhaustive, such as miRNA and lncRNA studies.

## Glossary

GABA: γ-aminobutyric acid
UGT: UDP-glucuronosyhransferase
miRNA: micro-RNA
lncRNA: long non-coding RNA
RISC: RNA-induced silencing complex
EMT: epithelial-mesenchymal transition
MIEN1: migration and Invasion Enhancer 1
NMDAR: N-methyl-D-aspartate receptor
circRNA: circular RNA
SOD2: superoxide dismutase 2
FOXM1: forkhead box M1
SFA_FA: saturated fatty acids to total fatty acids
DDP: cisplatin
PTX: Paclitaxel
5-FU: 5-Fluorouracil
TME: The tumor microenvironment
ECM: extracellular matrix
HIF: hypoxia-inducible factor
ROS: reactive oxygen species
HMGB1: The high mobility group protein B1
COX: cyclooxygenase
BM: basement membrane
MMP: metalloproteinase
ADAM: disintegrin and metalloprotease
LRR: local-regional recurrence
IDC: invasive ductal carcinoma
TM: total mastectomy
BCS: breast conservative surgery
PB-RA: paravertebral block-regional anesthesia
INHA-GA: inhalational general anesthesia.
